# Successful Resolution of Eruptive Xanthomas in Severe Hypertriglyceridemia With Plasmapheresis

**DOI:** 10.7759/cureus.79293

**Published:** 2025-02-19

**Authors:** Arūnas Petkevičius, Robertina Cibulskaitė, Ugnė Janonytė, Jurgita Makštienė, Birutė Žilaitienė

**Affiliations:** 1 Skin and Venereal Diseases, Hospital of Lithuanian University of Health Sciences (LSMU) Kauno klinikos, Kaunas, LTU; 2 Skin and Venereal Diseases, Lithuanian University of Health Sciences (LSMU) Medical Academy, Kaunas, LTU; 3 Pathology, Hospital of Lithuanian University of Health Sciences (LSMU) Kauno klinikos, Kaunas, LTU; 4 Endocrinology, Hospital of Lithuanian University of Health Sciences (LSMU) Kauno klinikos, Kaunas, LTU; 5 Institute of Endocrinology, Lithuanian University of Health Sciences (LSMU) Medical Academy, Kaunas, LTU

**Keywords:** eruptive, hypertriglyceridemia, plasmapheresis, xanthoma, xanthomatosis

## Abstract

Eruptive xanthomatosis, characterised by yellowish skin papules, is often associated with hypertriglyceridemia and can signal underlying systemic conditions such as uncontrolled diabetes. Here, we present the case of a 47-year-old woman with eruptive xanthomas and severe hypertriglyceridemia. Taking into consideration the multiple comorbidities, including type 2 diabetes, obesity, and a history of acute pancreatitis, the patient was treated with six courses of plasmapheresis, leading to complete resolution of the lesions and normal triglyceride (TG) levels.

## Introduction

Eruptive xanthomas serve as a clinical marker of severe hypertriglyceridemia and often indicate underlying systemic conditions such as uncontrolled diabetes and obesity [1]. Prompt recognition of eruptive xanthomas is crucial for clinicians to prevent complications like pancreatitis and cardiovascular disease. However, drug therapy for hypertriglyceridemia should be deferred when triglyceride (TG) levels exceed 11.3 mmol/l, as medications used to model lipid profile are less effective in such cases [2]. We propose that plasmapheresis could be considered for early management of severe hypertriglyceridemia, in cases with a high risk of pancreatitis development.

## Case presentation

A 47-year-old woman presented with a chronic, progressive papulous rash, situated on the extensor surfaces of the extremities and buttocks. In the patient's medical history, it was noted that she has been diagnosed with type 2 diabetes for the past 10 years. Initial treatment consisted of metformin and gliclazide. However, the last-known glycated haemoglobin (HbA1c) level was 11.5%, while the goal for majority non-pregnant adult diabetic patients is considered to be < 7%. The patient also had class 1 obesity, with a BMI of 32.6 kg/m^2^, diabetic polyneuropathy, secondary hypertension, and hepatic steatosis. It is also important to note that the patient was previously treated for acute pancreatitis, diagnosed four year after her type 2 diabetes diagnosis.

Upon dermatologic examination, she was found to have multiple yellowish-pink nontender papules 1-5 mm in diameter, resembling xanthomas. Lesions were located on the extensor surface of both arms, thighs, and buttocks, with some involvement of the abdomen (Fig. ​1).

**Figure 1:**
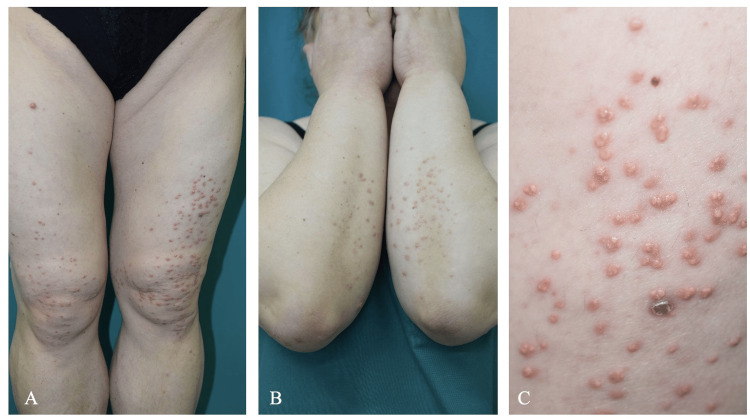
Xanthomas on the extensor surfaces of the extremities (A, B). Appearance of eruptions: yellowish pink papules 1-5 mm in diameter (C).

Laboratory results indicated severe hypertriglyceridemia at 36.64 mmol/l, elevated total cholesterol (TC) at 13.71 mmol/l, with low high-density lipoprotein cholesterol (HDL_C) at 0.66 mmol/l, low-density lipoprotein cholesterol (LDL_C) at 0.27 mmol/l, and a high atherogenicity index (AI) of 19.77. The values for the laboratory investigations are provided in Table 1 below.

**Table 1:** Laboratory investigations and reference ranges

Parameter	Result (mmol/L)	Reference Range (mmol/L)
Triglycerides (TG)	36.64	0-1.95
Total Cholesterol (TC)	13.71	0-5.2
High-Density Lipoprotein (HDL_C)	0.66	>1.55
Low-Density Lipoprotein (LDL_C)	0.27	0-2.59
Atherogenicity Index (AI)	19.77	0-3.5

**Figure 2:**
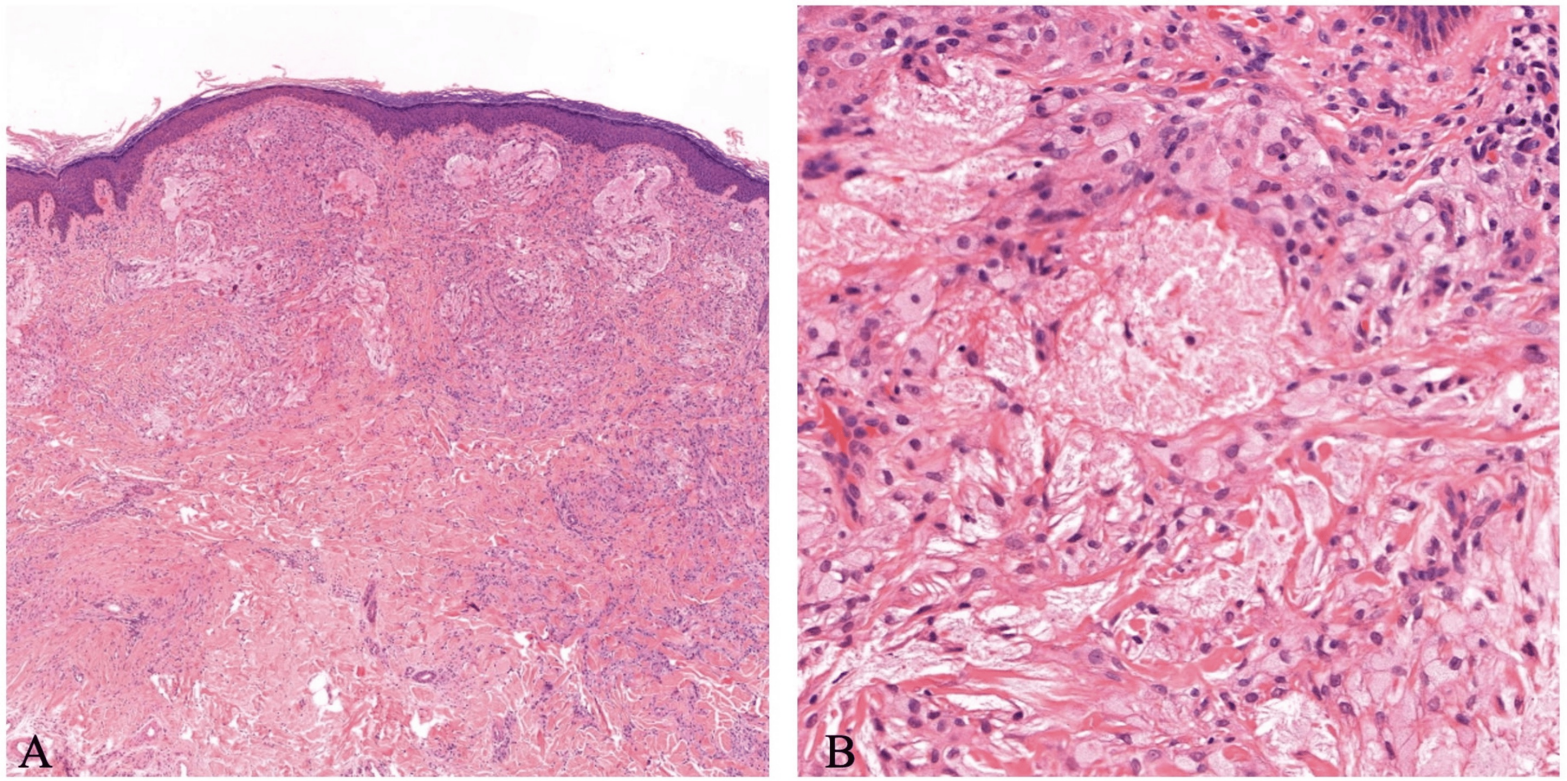
Macrophages are observed within the superficial and deep dermis, where a subset forms clusters of foam cells with cytoplasm laden with lipid inclusions (A, B). Representative microscopic images at ×100 (A) and ×400 (B).

A skin biopsy sample obtained from the papules revealed xanthomatous characteristics, with macrophages observed in both the superficial and deep dermis. In these areas, a subset of macrophages formed clusters of foam cells, their cytoplasm laden with lipid inclusions (Figure 2). Thus, we established the diagnosis of suspected eruptive xanthomatosis.

**Figure 3:**
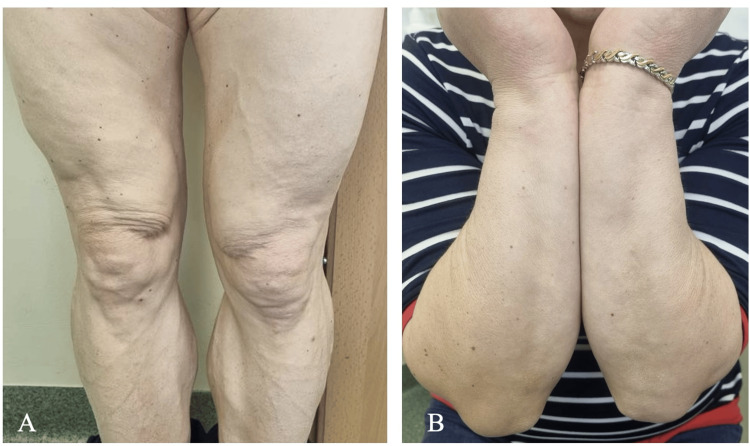
Complete resolution of the rash on the extremities (A, B), observed during a follow-up half a year after inpatient treatment

Due to severe hypertriglyceridemia and inadequately controlled diabetes, the patient was referred to the endocrinology department. During inpatient care, in addition to metformin intensive insulin therapy was started and improvement of glycemic control was achieved. Considering the previous case of acute pancreatitis in the patient history, six courses of plasmapheresis at 24- to 48-hour intervals were scheduled. Approximately 4,800 ml of blood was removed and filtered during the following week. This treatment plan resulted in a decrease in the TG level (8.05 mmol/l) and overall improvement of the lipid profile (TC: 4.32 mmol/; HDL_C: 0.73 mmol/l; LDL_C: 1.09 mmol/l; AI: 4.92). No complications associated with plasmapheresis were noted. The patient was prescribed statins (atorvastatin 40 mg per day), educated on proper diabetes management, and then discharged from the hospital. A follow-up after six months revealed a complete resolution of the rash, with no recurrence of the lesions (Figure 3).

## Discussion

Underlying causes and pathophysiology of eruptive xanthomas

The manifestation of xanthomatosis is typically associated with the state of hyperlipoproteinemia, as lipoproteins are able to pass through vascular endothelial cells and accumulate in the dermis, subcutaneous tissues, or tendons. Tissue macrophages then phagocytose these lipid deposits, leading to the formation of "foam cells" in xanthomas [3,4]. Xanthomas can be categorised as primary, arising from genetic mutations causing defective apolipoproteins, or secondary, resulting from acquired systemic disorders [5]. Additionally, the development of eruptive xanthomas is thought to be driven by hypertriglyceridemia and chylomicronemia, stemming from genetic deficiencies (e.g., familial chylomicronemia, mixed hyperlipidemia), or association with factors like obesity, excessive alcohol intake, and uncontrolled diabetes [1]. 

Clinical presentation of eruptive xanthomas

Clinical variants of xanthomas include papulonodular (tendinous, eruptive, tuberous), planar (diffuse planar, disseminated, palmar striated, and xanthelasma) and verruciform xanthomas [4,6]. Eruptive xanthomas present as yellowish skin papules, typically on extensor surfaces of the extremities and buttocks, often linked with severe hypertriglyceridemia [6,7]. They can be accompanied by systemic manifestations of lipid disorders, such as pancreatitis, as well as be a sign of poorly controlled diabetes [8,9]. Early lesions start to form within three weeks following a plasma TG increase and can be associated with pruritus, tenderness and Koebner phenomenon [4].

Diagnostic evaluation of eruptive xanthomas

Diagnosing xanthomas involves evaluating the type and cause through patient history, thorough physical examination, and laboratory studies [5]. Skin biopsy may be necessary for unclear cases and differential diagnosis. For example, a histopathological picture of an inflammatory infiltrate with foam cells and lipid deposits, should indicate the diagnosis of eruptive xanthoma, and help exclude sebaceous hyperplasia, juvenile xanthogranuloma, nodular basal cell carcinoma, etc. [9]. However, when metabolic syndrome features are present alongside TG levels exceeding 11.3 mmol/l, a biopsy may be unnecessary, as prompt recognition reduces the time to diagnosis and treatment, decreasing the risk of complications [10]. Laboratory evaluation should include a fasting lipid panel to assess dyslipidemia. Based on patient history and symptoms, further testing could be done to evaluate symptomatic coronary atherosclerosis, diabetes, thyroid, liver or renal diseases. This could be initially assessed by plasma atherogenicity level, fasting glucose, HbA1C, liver function, thyroid-stimulating hormone, and renal function tests [3,6]. It has been also suggested that such noninvasive diagnostic methods as dermoscopy and reflectance confocal microscopy (RCM) could be used to facilitate early diagnosis of xanthomas. For example, Yan et al. have documented such dermatoscopic characteristics as yellow-orange areas indicative of dermal xanthomatised cell accumulation, and features resembling foamy histiocytes on RCM, supporting the diagnosis of eruptive xanthoma [11]. To summarise, Marogi et al. propose steps for prompt eruptive xanthoma diagnosis: (i) perform a thorough skin examination; (ii) recognise the link between metabolic syndrome and severe hypertriglyceridemia; (iii) evaluate the four Ds: diet/lifestyle, drugs/medications, diseases/disorders of metabolism; (iv) initiate timely management [10].

Management and treatment of eruptive xanthomas

Management of underlying medical conditions is key in treating eruptive xanthomas. This includes a low-fat diet and medication to control TG level (e.g., statins, omega-3 fatty acids, fibrates and niacin) [12]. As lipid levels normalise, eruptive xanthoma lesions diminish gradually, and other treatment options (surgical, laser, or cryosurgical) are typically not required [9]. However, when TG levels surpass 11.3 mmol/l, the risk of hypertriglyceridemia-induced acute pancreatitis (HTGP) rises markedly, due to cytotoxic injury caused by free fatty acids from TG hydrolysis [13]. Guidance regarding the optimal choice of treatment in these cases is lacking. Management of HTGP usually involves pain control, fluid resuscitation, dietary fat restriction or even nil per os to reduce serum TG levels to <5.6 mmol/l. In patients with HTGP and concerning features, such as severe systemic inflammation or organ dysfunction, plasmapheresis is suggested until TG levels drop below 5.6 mmol/l. Although plasmapheresis significantly reduces TG, evidences supporting better clinical outcomes when using plasmapheresis for HTGP are insufficient [14,15]. If plasmapheresis is not available, initiating therapy with intravenous regular insulin is recommended, with close monitoring of TG levels and glucose supplementation as needed [14]. The role of heparin to this day remains controversial as it may contribute to further depletion of lipoprotein lipase [16].

## Conclusions

This case supports the use of plasmapheresis for the resolution of eruptive xanthomas, as it effectively corrects lipid levels in cases of severe hypertriglyceridemia. Additionally, plasmapheresis may serve as a valuable option for preventing complications. One such complication is acute pancreatitis, which is associated with severely elevated TG levels.
